# Systems Biology and Birth Defects Prevention: Blockade of the Glucocorticoid Receptor Prevents Arsenic-Induced Birth Defects

**DOI:** 10.1289/ehp.1205659

**Published:** 2013-01-03

**Authors:** Bhavesh K. Ahir, Alison P. Sanders, Julia E. Rager, Rebecca C. Fry

**Affiliations:** Department of Environmental Sciences and Engineering, Gillings School of Global Public Health, University of North Carolina at Chapel Hill, Chapel Hill, North Carolina, USA

**Keywords:** birth defects, comparative toxicogenomic database, glucocorticoid receptor pathway, metals, systems biology

## Abstract

Background: The biological mechanisms by which environmental metals are associated with birth defects are largely unknown. Systems biology–based approaches may help to identify key pathways that mediate metal-induced birth defects as well as potential targets for prevention.

Objectives: First, we applied a novel computational approach to identify a prioritized biological pathway that associates metals with birth defects. Second, in a laboratory setting, we sought to determine whether inhibition of the identified pathway prevents developmental defects.

Methods: Seven environmental metals were selected for inclusion in the computational analysis: arsenic, cadmium, chromium, lead, mercury, nickel, and selenium. We used an *in silico* strategy to predict genes and pathways associated with both metal exposure and developmental defects. The most significant pathway was identified and tested using an *in ovo* whole chick embryo culture assay. We further evaluated the role of the pathway as a mediator of metal-induced toxicity using the *in vitro* midbrain micromass culture assay.

Results: The glucocorticoid receptor pathway was computationally predicted to be a key mediator of multiple metal-induced birth defects. In the chick embryo model, structural malformations induced by inorganic arsenic (iAs) were prevented when signaling of the glucocorticoid receptor pathway was inhibited. Further, glucocorticoid receptor inhibition demonstrated partial to complete protection from both iAs- and cadmium-induced neurodevelopmental toxicity *in vitro*.

Conclusions: Our findings highlight a novel approach to computationally identify a targeted biological pathway for examining birth defects prevention.

Toxic metals are ubiquitous in the environment and are known to cause detrimental health effects. Exposure to toxic metals during the prenatal period is of particular concern, and exposure can occur as a result of diet, drinking water, airborne particles, consumer products, and certain occupational environments (Tabacova 1986). Evidence suggests that many toxic metals indeed cross the placental barrier (Al-Saleh et al. 2011; Casey and Robinson 1978; Concha et al. 1998; Guo et al. 2010; Rudge et al. 2009) and are thus likely to have a detrimental impact on the developing fetus.

The focus of the present study was to select seven high-priority toxic metals and/or metalloids on the basis of their presence in the environment and their known or suspected roles as developmental toxicants. The following metals were selected for study: cadmium (Cd), chromium (Cr), inorganic arsenic (iAs), lead (Pb), mercury (Hg), nickel (Ni), and selenium (Se). Four of these—Cd, Hg, iAs, and Pb—are ranked in the top 10 most hazardous substances by the Agency for Toxic Substances and Disease Registry (ATSDR 2010). Several of these metals have been associated with structural malformations and/or neural tube defects in animal models (Chaineau et al. 1990; Fernandez et al. 2004; Gilani and Marano 1980; Gruenwald 1958; Hovland et al. 1999; Messerle and Webster 1982; Tabocova et al. 1996; Thompson and Bannigan 2001). In addition to the toxicological data, several epidemiological studies have also examined the relationship between metal exposure and early life outcomes in infants. For example, prenatal exposure to iAs in drinking water has been associated with adverse pregnancy outcomes such as spontaneous abortion, stillbirth, preterm birth (Ahmad et al. 2001), and congenital malformations (Kwok et al. 2006; Zierler et al. 1988).

Clearly, there is public health concern surrounding environmental exposure–mediated birth defects, which is supported by both epidemiological and animal-based evidence. Still, the underlying pathophysiological mechanisms linking prenatal exposures to developmental disorders remain largely unknown. In the present study we set out to test the hypothesis that biological pathways that mediate metal-induced birth defects could be revealed by identifying common signaling pathways that integrate both metal- and development-associated genes. We applied a systems biology approach coupled with a teratogenic experimental strategy.

Specifically, the research framework included *a*) the identification of gene–contaminant relationships from a comparative toxicogenomics database; (*b*) the prediction of biological pathways associated with metals exposure and developmental disorders; and *c*) laboratory-based validation of the *in silico* pathway prediction. This novel computational approach was applied to the seven metals of interest and resulted in the prediction that the glucocorticoid receptor (GR) signaling pathway may be a key mediator that is highly associated with four of the selected metals: Cd, Hg, iAs, and Se. Focusing on this pathway, we used the *in ovo* chick embryo culture model to demonstrate that structural malformations induced by one of the metals, iAs, can be prevented through blockade of the GR signaling pathway. In addition, we used an *in vitro* micromass (MM) culture assay to demonstrate that neurodevelopmental toxicity induced by iAs and Cd was partially or completely prevented by blocking the pathway. Our results provide evidence for a novel systems biology strategy by which biological pathways can be predicted and subsequently tested to increase our understanding of pathophysiological mechanisms related to birth defects.

## Materials and Methods

*Identifying metal-associated genes.* To identify genes known to be associated with the metals of study, we used the Comparative Toxicogenomics Database (CTD 2011; Davis et al. 2011). The CTD is a manually curated toxicogenomic database. At the time of analysis, it included > 178,000 interactions between 4,980 chemicals and 16,182 genes/proteins in 298 species. It contains 8,900 gene/protein–disease direct relationships and 5,600 chemical–disease relationships (CTD 2011; Davis et al. 2011). We used the CTD Batch Query tool (CTD 2011) to retrieve all curated chemical–gene/protein interactions for each of the seven selected metals: Cd, Cr, Hg, iAs, Ni, Pb, and Se. In addition, the CTD was used to identify genes/proteins associated with phenytoin, a well-known human teratogen (Buehler et al. 1990), which served as a positive control for the *in ovo* experiments.

*Identifying metal-associated genes with roles in development.* Once metal-associated genes/proteins were identified using the CTD database, we performed biological function enrichment analysis using Ingenuity Pathway Analysis (IPA) software (Ingenuity Systems, Redwood City, CA). Specifically, genes with known involvement in embryonic development and developmental disorders were identified and referred to as “development” associated.

*Predicting pathways involved in metal-induced developmental disorders.* Molecular networks related to metal-associated genes involved in development were identified using IPA. This knowledge database provides a collection of gene-to-phenotype associations, molecular interactions, regulatory events, and chemical knowledge accumulated to develop a global molecular network. In IPA, metal-associated genes were mapped to their global molecular networks, and networks integrating proteins encoded by the metal- and development-associated genes were algorithmically generated based on their connectivity. Pathway enrichment analysis was performed to identify canonical pathways significantly associated with constructed networks. Statistical significance of each constructed network was evaluated using Fisher’s exact test.

In ovo *whole chick embryo culture.* The most significant canonical pathway identified through network analysis was ranked and validated for its involvement in embryonic development using the chick embryo model. Specifically, we used *in ovo* whole chick embryo culture assay, a well-established model for teratogenicity assessment (Kucera et al. 1993), to test the computational prediction that the GR signaling pathway is involved in metal-induced developmental disorders. All experimental procedures were conducted on embryos < 10 days of age and thus were exempt from oversight by the University of North Carolina Institutional Animal Care and Use Committee. We obtained fertilized white leghorn chicken eggs from Charles River Laboratories (North Franklin, CT, USA). Eggs were randomly selected and divided into seven different treatment groups immediately before incubation. The treatment groups were as follows: control [phosphate-buffered saline (PBS) only]; vehicle control (0.1% ethanol); phenytoin, a positive control for neural tube defects (Fisher Scientific); iAs as sodium arsenite (iAs^3+^; Sigma-Aldrich, St. Louis, MO); cortexolone, a GR inhibitor (Fisher Scientific); phenytoin plus cortexolone; and iAs^3+^ plus cortexolone. We selected the concentration of cortexolone on the basis of previous studies (Harlow et al. 1987; Turnell et al. 1974).

Ten to 12 embryos were examined per treatment group in four independent biological replicates. Eggs were incubated at 100°F at a relative humidity of 55%, with the day on which the eggs were incubated counted as day 0. The eggs were dosed on day 3 of incubation. First, the eggs were swabbed with 70% ethanol and the blunt end of the eggs was stuck with forceps to make a small hole, following an established protocol (Kucera et al. 1993; Memon and Pratten 2009). Eggs were injected with 100 µL of either PBS alone, vehicle (0.1% ethanol), phenytoin (400 µM), iAs^3+^ (0.1 µM, or 7.5 ppb), cortexolone (2 µM), phenytoin plus cortexolone, or iAs^3+^ plus cortexolone, and then incubated until day 6. On day 6, embryos were removed and analyzed for gross malformations and scored according to established morphological scoring criteria (described by Memon and Pratten 2009) [see Supplemental Material, Table S1 (http://dx.doi.org/10.1289/ehp.1205659)]. Only viable embryos, defined by the presence of a beating heart and visibly circulating blood in the embryo and yolk sac, were included and assigned morphological scores. Crown–rump length was taken as a measure of embryo growth.

Data were analyzed using Graphpad Prism 5 software (Graphpad Software Inc., San Diego, CA). To assess the morphological scoring criteria (vitelline circulation, flexion, heart, brain, gross facial deformities, and limbs) between exposure groups, we used Kruskal-Wallis one-way analysis of variance (ANOVA) followed by Mann-Whitney *U*-test for nonparametric data. The statistical process was carried out as described by Hewitt et al. (2005). The means ± SEs of the morphological scores were determined for the four independent experiments. Embryo growth, as measured by crown–rump length, was analyzed using parametric statistical tests (ANOVA followed by Dunnett’s post hoc test). Statistical significance was set at *p* < 0.05.

*Quantitative reverse-transcription polymerase chain reaction (qRT-PCR).* We assessed the gene expression levels of two transcription factors, *NF-*κ*B1* (nuclear factor kappa B1) and *AP1* (activating protein 1). *AP1* comprises *c-FOS* and *c-JUN,* and these genes were assessed relative to glyceraldehyde 3-phosphate dehydrogenase (*GAPDH*) using qRT-PCR; primer sequences are shown in Supplemental Material, Table S2 (http://dx.doi.org/10.1289/ehp.1205659). Total RNA was isolated from the whole head regions of treated and untreated embryos (*n* = 3 embryos/group) using an RNeasy® Mini Kit (QIAGEN, Valencia, CA). RNA was quantified with the NanoDrop^TM^ 1000 Spectrophotometer (Thermo Scientific, Waltham, MA), and integrity was verified with a model 2100 bioanalyzer (Agilent Technologies, Santa Clara, CA). Fold changes between the treatment group and the control group were calculated using delta-delta cycle threshold (ΔΔCt) values and normalized with *GAPDH* as a housekeeping gene. Statistical significance of the transcript levels between treatment groups versus the control group was calculated using an unpaired *t-*test.

*Chick embryo midbrain micromass (MM)culture* in vitro *assay.* We further evaluated the role of the GR pathway as a mediator of metal-induced neurodevelopmental toxicity using the *in vitro* chick embryo MM culture assay. The midbrain MM culture assay is a well-accepted method to screen for developmental toxicity of compounds, and was performed according to previously established protocols (Flint and Orton 1984; L’Huillier et al. 2002). Midbrains were removed from chick embryos on day 6 and trypsinized to prepare single-cell suspensions in Ham’s F12 culture medium containing 10% fetal calf serum, 200 mM l-glutamine, penicillin (50 units/mL), and streptomycin (50 µg/mL). Cell density was estimated using a hemocytometer and adjusted to 1 × 10^6^ cells/mL. A total of 10 µL of the cell suspension was pipetted into the center of a collagen-coated well in a 96-well culture plate. After 24 hr, midbrain MM cultures were treated with iAs^3+^ (1, 2, or 5 µM), Cd as CdCl_2_ (1, 2, 5, or 8 µM), phenytoin (100 µM), cortexolone (2 µM), cortexolone plus iAs^3+^, cortexolone plus CdCl_2_, cortexolone plus phenytoin. The assay included positive and negative controls for cytotoxicity, namely, 5-fluorouracil (5-Fu) and penicillin G (PenG), respectively. Midbrain MM culture cytotoxicity was measured after 5 days in culture using the one-step resazurin reduction assay (O’Brien et al. 2000). Optical density was measured on a Synergy™ HT Multi-Mode Microplate Reader (BioTek, Winooski, VT), with an excitation filter 530 ± 25 nm and emission filter 590 ± 35nm indicating cell viability and metabolic activity. Statistical analysis was performed using Graphpad Prism 5 software. We used Student’s *t*-test for comparison between two groups and one-way ANOVA followed by an appropriate post hoc test (e.g., Dunnett or Bonferroni) for comparisons among groups. A *p*-value < 0.05 was considered statistically significant.

## Results

We used a novel approach to identify biological pathways that may mediate environmentally induced birth defects. Specifically, our strategy involved using the CTD to identify genes associated with environmental metals, filtering these genes for biological function related to birth defects and development, and examining the genes for known biological interactions in the cell [see Supplemental Material, Figure S1 (http://dx.doi.org/10.1289/ehp.1205659)].

*Metal- and development-associated genes identified.* The *in silico* approach first involved the identification of genes associated with seven metals: Cd, Cr, Hg, iAs, Ni, Pb, and Se. Genes with known association to the metals of interest were identified using the CTD [see Supplemental Material, Table S3 (http://dx.doi.org/10.1289/ehp.1205659)]. The CTD contains data on broad relationships between genes and environmental toxicants (we queried for metals), known effects of toxicants on gene expression, and genes encoding proteins that have altered metal-associated changes in protein abundance and protein activity. Thus, all genes identified had a known relationship with at least one of the seven metals: Cd (*n* = 518 genes), Cr (*n* = 175 genes), Hg (*n* = 334 genes), iAs (*n* = 1,880 genes), Ni (*n* = 637 genes), Pb (*n* = 774 genes), and Se (*n* = 1,616 genes) (see Supplemental Material, Tables S3 and S4). In addition, genes associated with phenytoin, a known teratogen, were identified as a positive control (*n* = 138) (see Supplemental Material, Tables S3 andS4).

The lists of metal-associated genes were subsequently filtered for genes/proteins with known relationships to organismal development and birth defects. Specifically, we performed biological function enrichment analysis; genes related to either “embryonic development” or “developmental disorders” were identified and extracted from the lists. The resultant numbers of genes that were identified as both metal- and development-associated ranged from 76 for Cr to 604 for iAs [see Supplemental Material, Tables S3 and S5 (http://dx.doi.org/10.1289/ehp.1205659)].

**Figure 1:**
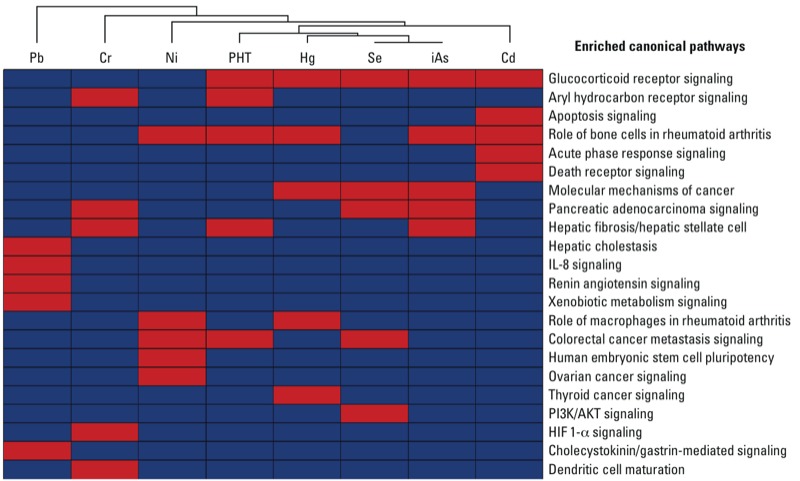
Canonical pathways associated with metal exposure and developmental disorders. A total of 22 canonical pathways found to be highly enriched in genes/proteins for at least one of the selected metals or phenytoin (PHT; the positive control), as identified in lists retrieved from the CTD. Colors denote pathways that were (red) or were not (blue) within the top five canonical pathways for that metal or phenytoin. Abbreviations: AKT, serine/threonine-specific protein kinase; HIF‑1α, hypoxia-inducible factor 1, alpha subunit; IL‑8, interleukin 8; PI3K, phosphoinositide 3-kinase.

*Predicting pathways influencing metal-induced developmental disorders.* Biological pathways that were enriched among the metal- and development-associated genes were algorithmically constructed. For each of the metals and for phenytoin, we selected the highest ranking (e.g., top five) canonical pathways. Because of some overlap, this resulted in a total of 22 unique canonical pathways [Figure 1; for a complete list of all top-ranking pathways and their *p*-values, see Supplemental, Table S6 (http://dx.doi.org/10.1289/ehp.1205659)]. The pathway analysis revealed that, in general, distinct pathways were highly enriched among genes associated with each of the metals (Figure 1). Still, many of the pathways were enriched between two or more metals. The GR signaling pathway was a high ranking canonical pathway for Cd, Hg, iAs, and Se. Notably, this indicates that genes associated with these four metal- and development-associated genes commonly mapped to the GR signaling pathway. Networks associated with phenytoin, a known teratogen, also showed significant enrichment for biological pathways that overlapped with those associated with metal exposure, including the GR signaling pathway. Other pathways of interest were identified as being related to the metal- and development-associated genes, such as the aryl hydrocarbon receptor (AHR) pathway identified for Cr and phenytoin (Figure 1).

To further establish environmental exposure–mediated network interactions, we performed a subsequent analysis using a combined list of all the metal- and development-associated genes together (*n* = 855 genes). Within the resulting metal-associated networks, the GR signaling pathway was the most significantly (*p* < 10^–6^) enriched pathway [see Supplemental Material, Figure S2 (http://dx.doi.org/10.1289/ehp.1205659)]. Many of the genes that encode proteins involved in the canonical GR signaling pathway have known association with numerous metals as defined in the CTD. For example, NF-κB1, a protein involved in the GR signaling pathway, has a known relationship to six of the seven metals (CTD 2011). Likewise, AP1 (composed of c-FOS and c-JUN) and c-JUN N-terminal kinase (JNK) are also associated with multiple metals (CTD 2011) (see Supplemental Material, Figure S2).

*Validation of computational prediction by the embryo culture assay.* Given the computational analysis predicting association of Cd, Hg, iAs, and Se with the GR signaling pathway, we set out to validate the findings using an *in ovo* whole chick embryo culture assay. Our hypothesis was that the GR mediates metal-induced developmental defects. iAs was prioritized for testing, and results were compared to those of phenytoin, a GR-dependent teratogen (Kay et al. 1990). A total of 10–12 embryos per treatment group were examined in four independent biological replicates.

**Figure 2:**
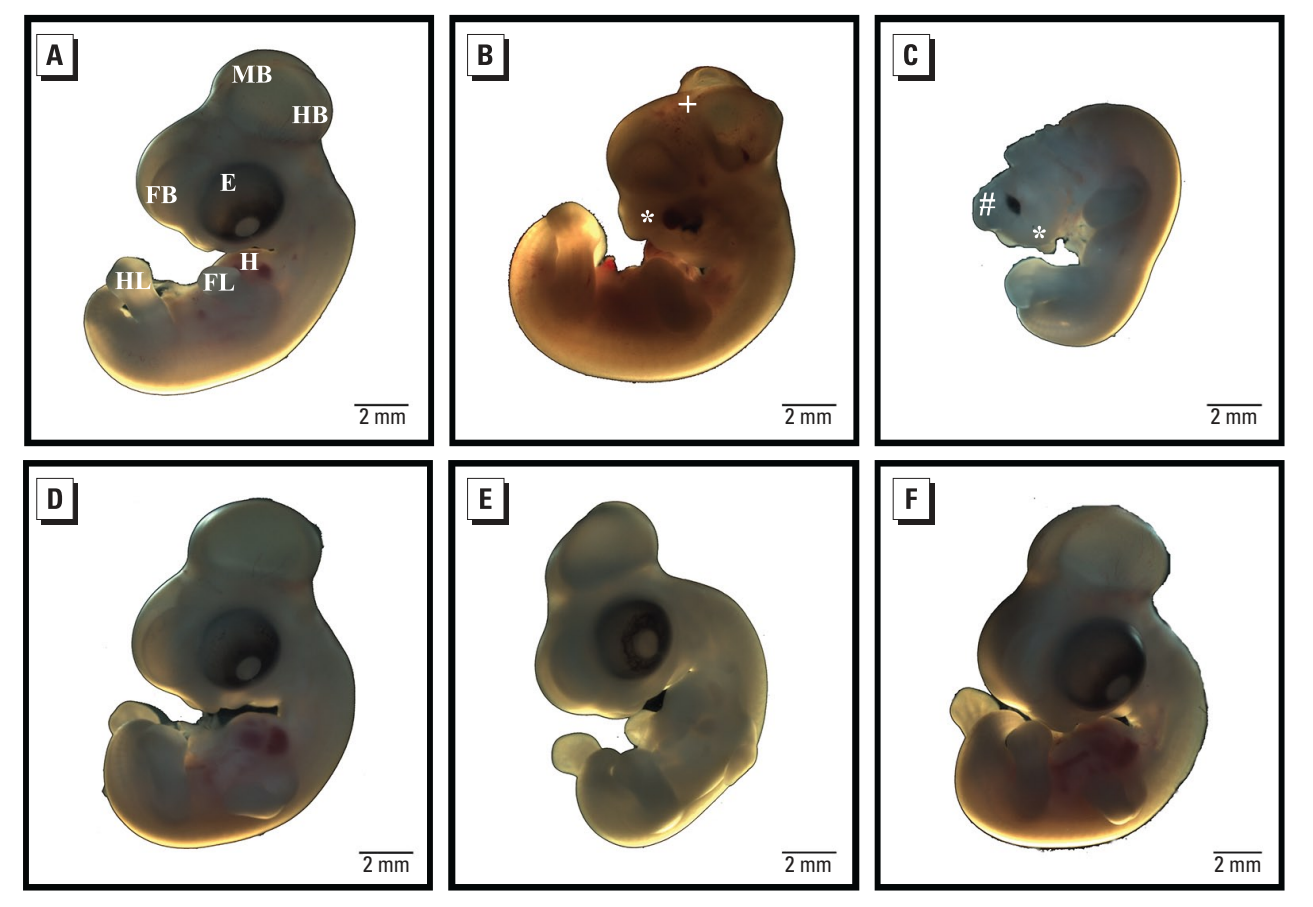
Photographs of representative embryos from each treatment group showing morphological features of chick embryos treated *in ovo*. Developmental end points were assessed between treatment and control groups on day 6. (*A*) Control (PBS-treated) embryo showing the development of forebrain (FB), midbrain (MB), hindbrain (HB), eye (E), heart (H), fore limb buds (FL), and hind limb buds (HL). (*B*) Phenytoin (PHT)-treated embryo exhibiting an abnormal head shape, failure of closure of the anterior part of the neural tube (+), and craniofacial defects (*). (*C*) iAs^3+^-treated embryo exhibiting craniofacial and anterior neural tube defects (anencephaly) (#). Embryos treated with (*D*) cortexolone (CX), (*E*) PHT plus CX, or (*F*) iAs^3+^ plus CX (*F*). Bar = 2 mm.

Phenytoin, the positive teratogenic control, induced malformations in 56% of the embryos, and iAs induced malformations in 59% (Table 1). No phenotypic abnormalities were observed in embryos treated with PBS or the vehicle control (Figure 2A, Table 1). For phenytoin and iAs, observed morphological defects included abnormalities in the head fold region, microcephaly, anterior neural tube defects, and gross facial deformities (Figure 2B,C). The craniofacial defects included altered optic and beak development and malformed facial arches. The *in ovo* exposures of chick embryos to iAs and phenytoin also induced lethality in 24% of the embryos (Table 1).

**Table 1:** Frequency of abnormal embryos.

Treatment group	Embryo explants (n)	Dead embryos (n)	Surviving embryos (n)	Normal [n (%)]	Abnormal [n (%)]
Control	42	1	41	41 (100)	0
Vehicle	42	3	39	39 (100)	0
Phenytoin	42	10	32	14 (44)	18 (56)
iAs	42	10	32	13 (41)	19 (59)
Cortexolone	42	2	40	40 (100)	0
Phenytoin plus cortexolone	42	8	34	34 (100)	0
iAs plus cortexolone	42	5	37	37 (100)	0

To specifically test the *in silico* prediction that the GR signaling pathway mediates the occurrence of structural birth defects caused by iAs, the GR pathway was blocked using the inhibitor cortexolone. Embryos exposed to cortexolone alone or to phenytoin plus cortexolone had no gross structural malformation and developed normally (Figure 2D,E, Table 1). Suprisingly, embryos exposed to iAs plus cortexolone showed no gross structural malformations and displayed normal growth parameters (Figure 2F, Table 1).

**Figure 3:**
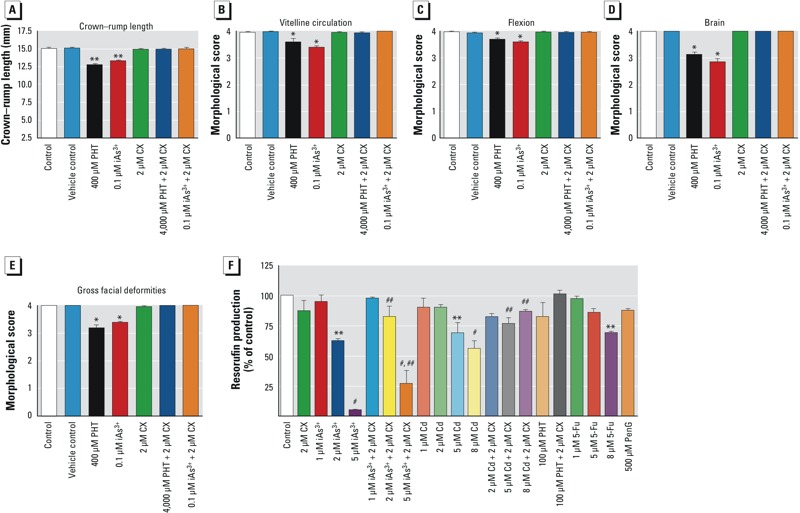
Embryonic growth, morphological scores, and toxicity of chemical compounds in whole chick embryos cultured *in ovo* (incubation day 3–6). Crown–rump length (*A*), vitelline circulation (*B*), flexion (*C*), brain (*D*), and gross facial deformities (*E*) of embryos treated with PBS (control), vehicle control (0.1% ethanol), phenytoin (PHT), iAs^3+^, cortexolone (CX), PHT plus CX, or iAs^3+^ plus CX. (*F*) Effects of iAs^3+^ and Cd (CdCl_2_) on cell viability of midbrain MM cultures 5 days after exposure; positive and negative controls for cytotoxicity were 5-Fu and PenG, respectively. Data represent the means ± SEs from four independent experiments (*n *= 4). *p < 0.05, **p < 0.01, and ^#^*p* < 0.001 compared with control and vehicle groups. ^##^*p *< 0.05 compared with 2 and 5 µM iAs^3+^ alone or 5 and 8 µM Cd alone.

Embryo growth (crown–rump length) was significantly (*p* < 0.05) decreased in phenytoin-exposed and iAs-exposed embryos compared with controls, whereas cortexolone-treated embryos developed normally and were comparable to the control group (Figure 3A). In addition, statistical analyses of other morphological scoring criteria showed a significant difference (*p* < 0.05) between iAs or phenytoin and the control groups for vitelline circulation, flexion of the embryo, brain development, and craniofacial development (Figure 3B–E). No gross malformations were detected in heart or limbs for any treatment group [see Supplemental Material, Figure S3A,B (http://dx.doi.org/10.1289/ehp.1205659)].

*Mediation of iAs-induced gene expression changes by GR.* To validate that iAs influences gene expression via the GR pathway in the developing chick, we performed qRT-PCR. We assessed the expression levels of two transcription factors, *NF-*κ*B1* and *AP1*, in the head regions of the embryos. *NF-*κ*B1* (fold change = 3.85) and *c-FOS* (fold change = 5.66) were up-regulated in embryos treated with iAs (0.1 µM) [see Supplemental Material, Figure S4A,B (http://dx.doi.org/10.1289/ehp.1205659)]. Embryos treated with iAs plus cortexolone showed restored levels of *NF-*κ*B1* (*p* < 0.01) and *c-FOS* (*p* < 0.05) (see Supplemental Material, Figure S4A,B). In phenytoin-treated embryos, gene expression levels of *NF-*κ*B1* and *c-JUN* were not significantly changed, but *c-FOS* expression was increased. Surprisingly, cortexolone did not block the effects of phenytoin on the expression level of *c-FOS*. These results suggest that other genes/proteins in the GR signaling pathway may influence phenytoin and cortexolone and their interactions in this model system. The increased *c-JUN* expression level was not statistically significant in any of the treatment groups (see Supplemental Material, Figure S4C).

*Mediation of iAs and Cd-induced neurodevelopmental toxicity by the GR pathway.* The GR pathway was predicted to mediate the response to Cd, Hg, iAs, and Se. Thus, as a further test of the role of the GR in mediating metal-induced neurodevelopmental toxicity, we chose to test Cd as well as iAs. Embryonic chick midbrain MM cultures were exposed to iAs (1–5 µM), Cd (1–8 µM), phenytoin (100 µM), or cortexolone (2 µM). We used 5-Fu and PenG, respectively, as positive and negative controls for cytotoxicity. The 8 µM concentration of 5-Fu resulted in a significant (*p* < 0.01) reduction in cytotoxicity (Figure 3F). This suggests that differentiating midbrain MM cultures were susceptible to the toxic effects of 5-Fu at this concentration. As expected, PenG was not cytotoxic to the midbrain MM cultures. Exposure of the cultures to phenytoin or phenytoin plus cortexolone did not result in any cytotoxicity. This is consistent with the results of Regan et al. (1990), who observed no cytotoxic effects in primary neuronal cells treated with this concentration of phenytoin. We observed a significant decrease in the viability of embryonic midbrain cells treated with 2 and 5 µM iAs or 5 and 8 µM Cd (Figure 3F). Cortexolone-treated cells, with inhibited signaling of the GR pathway, were protected from cytotoxicity induced by 2 µM iAs. At the highest iAs concentration (5 µM), midbrain MM cultures were partially protected from iAs-induced cytotoxicity. Cortexolone completely protected the midbrain cells from the cytotoxic effects of Cd (Figure 3F).

## Discussion

Congenital malformations are the leading cause of infant mortality. An estimated 120,000 infants are born with severe congenital malformations each year in the United States (Brent 2004). Of all the congenital malformations, 60–70% are caused by unknown environmental and/or genetic causes (Moore and Persaud 1998). Thus, there is a need for effective methods to identify biological pathways that are pathophysiologically related to birth defects. There is great promise that systems biology can be used to identify biological pathways that relate environmental contaminants with human development/diseases (Chuang et al. 2010) and birth defects (Sperling 2011).

In the present study, we developed an *in silico* strategy to aid in the understanding of metal-induced birth defects. To investigate the relationship between metal exposures and birth defects, we applied this novel approach whereby genes associated with seven selected metals were identified using the CTD. To identify genes with any known relationship to birth defects, the CTD-retrieved gene/protein data sets were then filtered for only those genes with known associations to embryonic development or developmental disorders. Biological pathways associated with developmental disorders were further elucidated using a systems-level analysis.

Although other studies have used the CTD to identify metal-perturbed pathways (Davis et al. 2008; Mattingly et al. 2009), we introduced a health outcome filter to enrich for relationships to structural birth defects that may result from exposure to toxic metals. The metal- and development-associated genes were highly enriched for 22 canonical pathways, many of which were common to multiple metals. For example, the GR signaling pathway was identified as a top ranking pathway for Cd, Hg, iAs, and Se. In addition, the GR pathway was also generally enriched in the genes/proteins identified for Cr, Ni, and Pb. This finding is supported by other reports that metals, including Cd, Cr, Hg, iAs, Pb, and Se, influence signaling of the GR signaling pathway (Webster Marketon and Sternberg 2010). Although these metals have been shown to be associated with the GR, the role of this pathway in mediating metal-induced developmental defects is understudied. In addition to the GR pathway, other pathways, such as that mediated by AHR, were significantly enriched for Cr and for phenytoin. These findings suggest other biological pathways that warrant future follow-up. These results may also provide a means by which biological pathways that mediate the developmental defects of metals are prioritized for study.

In general, structural birth defects are believed to result from complex mechanisms, including multiple genes and signaling pathways (Mitchell 2005). The GR pathway exemplifies this complexity and is composed of pro-inflammatory cytokines, enzymes, cell adhesion molecules, and transcription factors such as NF-κB1 and AP1. Our results show that many of the genes/proteins involved in the GR pathway are enriched for interactions of two or more of the metals. For example, NF-κB1 is associated with Cd, Hg, iAs, Ni, Pb, and Se (CTD 2011).

We postulated that the GR signaling pathway may be a biological pathway that associates metals with resultant structural birth defects. To test this computational prediction, we prioritized one of the selected study metals for testing using the whole chick embryo culture *in ovo* model. We selected iAs for further investigation because it is a well-established developmental toxicant and is listed as the highest priority hazardous substance by the ATSDR (2010). iAs can readily cross the human placenta and accumulate in fetal neuroepithelium junction, thereby plausibly inducing various developmental defects (Wlodarczyk et al. 1996, 2006).

In the present study, we observed that low levels of iAs (0.1 µM, or 7.5 ppb) induced structural birth defects including microcephaly, anterior neural tube defects (anencephaly), and gross craniofacial defects in the chick embryo. These results are similar to those from previous studies that showed iAs-induced structural malformations in the chick (Han et al. 2011) and mouse embryo models (Chaineau et al. 1990; Tabocova et al. 1996). The iAs-induced malformations in mouse have also been shown to be associated with systems-level changes in gene expression (Robinson et al. 2011).

Although strong evidence demonstrates the teratogenic effects of iAs in animal models, debate remains as to the effects on human congenital malformation (DeSesso 2001; Golub et al. 1998; Holson et al. 2000). Potential epidemiologic associations relating iAs with birth defects have been reported in human populations (Kwok et al. 2006; Zierler et al. 1988). For example, iAs-associated low birth weight has been previously demonstrated in human populations (Hopenhayn et al. 2003). The iAs concentration used in the present study is within the 10 ppb (0.13 µM) maximum allowable level of arsenic in U.S. public drinking water supplies (U.S. Environmental Protection Agency 2012).

The mechanism by which iAs induces structural birth defects in animal models is largely unknown. Given the results of our computational analyses, we postulated that iAs may induce birth defects via the GR signaling pathway. To test this, we used the known GR inhibitor cortexolone. Cortexolone binds to the GR and leads to an altered conformational form of the GR complex, which is then transported into the nucleus (Kaiser et al. 1972). We observed that blockade of the GR pathway indeed prevents the iAs-induced craniofacial and neural tube defects. Others have reported that cortexolone successfully reduces the occurrence of phenytoin-induced birth defects in mouse embryos (Kay et al. 1990). Cortexolone also prevents GR-mediated teratogenicity (e.g., cleft palate, limb defects) in chick embryos (Jelinek et al. 1983; Pavlik et al. 1986). To our knowledge, no previous studies have examined GR blockade as a means for prevention of metal-induced birth defects.

Our results show a relationship among iAs, birth defects, and the GR pathway. iAs has a biphasic effect on GR function and disrupts GR-mediated transcription in a complex fashion. Specifically, very low doses of iAs (e.g., 0.01 µM, or 0.7 ppb) have been shown to alter the function of the GR as a transcription factor, enhancing GC induction of endogenous GR-regulated genes (Bodwell et al. 2004; Davey et al. 2007; Kaltreider et al. 2001). Studies have also shown that low doses of iAs (e.g., 0.1 µM, or 7.5 ppb) can interfere with hormone receptor binding and can act as a potent endocrine disruptor of hormone-mediated gene transcription by the GR (Bodwell et al. 2004, 2006; Davey et al. 2007; Kaltreider et al. 2001). Furthermore, iAs causes altered signaling via oxidative stress induction, which can damage DNA in cells by turning on heat shock protein production (Hughes 2002). Through these mechanisms, iAs may activate GR-mediated gene transcription. Several studies have demonstrated that iAs enhances NF-κB1 and AP1 DNA binding and induces stress responsive transcription factors that may play important roles in iAs-induced signal transduction, cell transformation, and apoptosis (Dong 2002; Drobna et al. 2003; reviewed by Zeng et al. 2005). In the present study, we examined the expression levels of GR-mediated *NF-*κ*B1* and *AP1* and found that iAs increased their expression levels in the head region of the deformed chick. Their expression levels were muted in the presence of cortexolone, indicating that iAs influences the expression of these genes via the GR pathway. It is important to note that phenytoin, the GR-specific control, failed to impact the expression of *NF-*κ*B1*, with only *c-FOS* showing a statistically significant change. These data suggest complex signaling responses to phenytoin that may act on other genes within the GR pathway.

GC-associated effects are mediated through the GR. Several possible mechanisms can cause the disruption of the GR signaling pathway, such as excessive amounts of GCs leading to intrauterine growth retardation and low birth weight (Drake et al. 2007). In the fetus, unmetabolized GCs appear to function as the active teratogenic agent (Goldman et al. 1978; Pratt 1985). GCs can also influence glycolysis via a GR-mediated mechanism (Loiseau et al. 1985). Evidence suggests that GC teratogenicity is a result of direct action on the embryo, which triggers a characteristic pattern of dysmorphogenesis via the biochemical and GC-mediated anti-inflammatory pathway (Kay et al. 1990; Pratt 1985).

**Figure 4:**
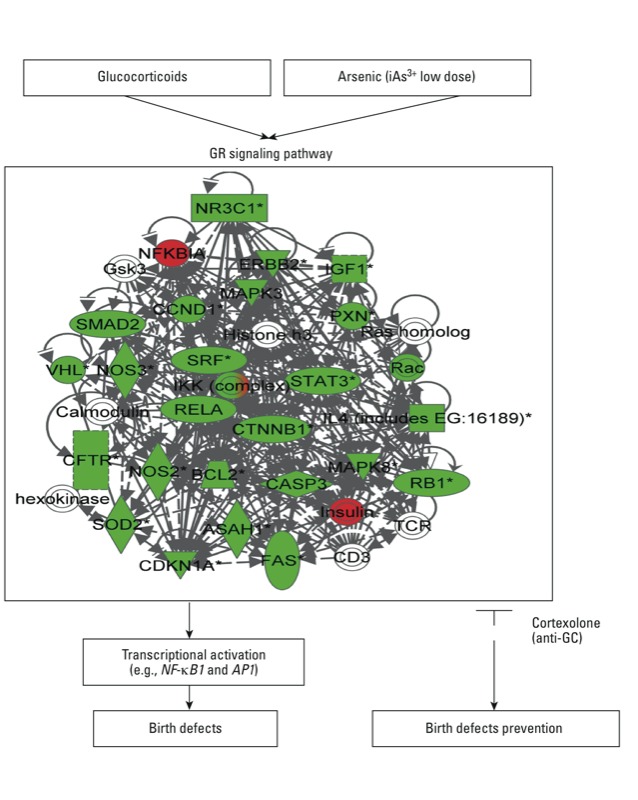
Proposed model for iAs-induced birth defects via the GR pathway. iAs appears to induce birth defects in the chick embryo as a result of signaling through the GR [also known as NR3C1 (nuclear receptor subfamily 3, group C, member 1)] pathway. Gene members of the GR pathway are shown in red (associated with embryonic development) or green (associated with developmental disorders). In embryos with inhibited signaling of the GR pathway via cortexolone, the iAs-induced birth defects are prevented.

The *in silico* results presented here indicate that not only iAs but other metals, such as Cd, may also act through the GR. To examine the links between the GR pathway and these two separate metals, we further assessed the toxicity of iAs or Cd in midbrain MM culture, an established method to assess the effects of developmental toxicants (L’Huillier et al. 2002). The results demonstrate that inhibition of GR signaling partially protected against iAs-induced cytotoxicity at the highest dose, while complete protection was observed at the lower doses. GR blockade completely protected against Cd-induced cellular toxicity. These data support the prediction that metals other than iAs also act via the GR pathway. Taken together, the results from this study show that iAs-induced structural birth defects are dependent on signaling through the GR pathway (Figure 4). These findings highlight an *in silico* method useful for the selection of a targeted biological pathway to test for birth defects prevention. The results suggest a plausible pathophysiological mechanism by which iAs alters the GR pathway to ultimately cause birth defects.

## Conclusions

We used a systems biology–based computational approach to determine that the GR pathway is a highly enriched pathway integrating a panel of metals (e.g., Cd, Hg, iAs, Se) with birth defect–associated genes. On the basis of the computational prediction, we used a GR inhibitor to demonstrate that iAs-induced structural malformations can be prevented in the chick embryo model. In addition, iAs- and Cd-induced neurodevelopmental toxicity were partially or completely protected via GR pathway inhibition assessed using the midbrain MM culture assay. Thus, these results illustrate the potential use of systems biology–based predictions in teratology and developmental toxicology research. We anticipate that this novel strategy can be employed to predict other biological pathways that mediate environmentally induced birth defects. Moreover, as applied to environmental metals, this type of cost-effective approach could be applied to a wide range of other environmental contaminants. These data provide novel information that may be useful in the prevention and treatment of metal-induced birth defects.

## Supplemental Material

(1.3 MB) PDF

(254 KB) XLS

(74 KB) XLS

(16 KB) XLS
