# Supplementary material for: Systems Biology and Birth Defects Prevention: Blockade of the Glucocorticoid Receptor Prevents Arsenic-Induced Birth Defects
Source: Environ Health Perspect. 2013 Jan 3;121(3):332–8. doi: 10.1289/ehp.1205659 (PMC3616967; doi:10.1289/ehp.1205659)
Supplement: (1.3 MB) PDF [file ehp.1205659.s001.pdf]

Supplemental Material

Systems Biology and Birth Defects Prevention: Blockade of the Glucocorticoid Receptor Prevents Arsenic-Induced Birth Defects

Bhavesh K. Ahir, Alison P. Sanders, Julia E. Rager, and Rebecca C. Fry

Supplemental Material

TABLE OF CONTENTS

Supplemental Material, Figure S1: An *in silico* approach was used to predict biological pathways influencing metal-associated development……………………………………………03

Supplemental Material, Figure S2: The glucocorticoid receptor (GR) signaling pathway is the most significant canonical pathway that associates metal exposure with birth defects…………04

Supplementary Material, Figure S3: Morphological scores and embryonic growth................05

Supplementary Material, Figure S4: Quantitative real time PCR gene expression analysis of *NF-κB1*and *AP1*………………………………………………………………………….06

Supplemental Material, Table S1: Morphological scoring based on Hamburger & Hamilton staging system....………………………...07

Supplemental Material, Table S2: List of primers for quantitative real-time RT-PCR analysis...08

Supplemental Material, Table S3: Selected metals of study………………………………..09

Supplemental Material, Table S4: The CTD curated metal-interacting genes and proteins (See Separate Excel file)...10

Supplemental Material, Table S5: The genes that were identified as metal-associated and involved in development. (See Separate Excel file)..11

Supplemental Material, Table S6: *p*-values of the enrichment of the top five canonical pathways associated with each designated metal and phenytoin (See Separate Excel file)........12

REFERENCES…………………………………………………………………….....13

Supplemental Material, Figure S1

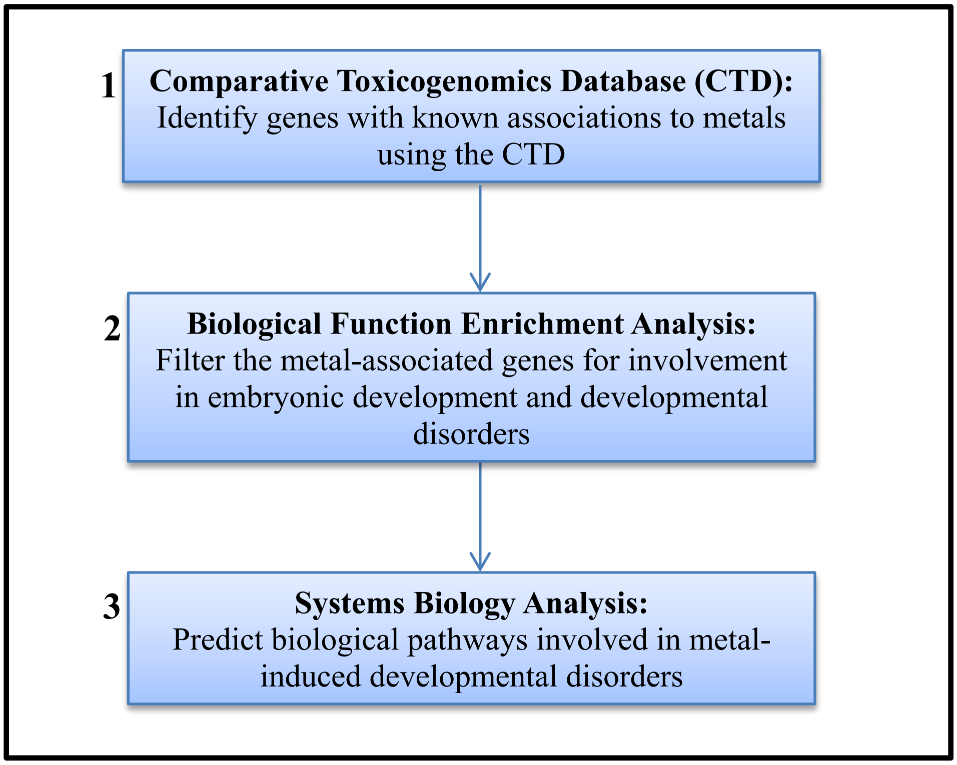

Supplemental Material, Figure S1: **An *in silico* approach was used to predict biological pathways influencing metal-associated development.** A three step process was used to: **(1)** identify genes associated with environmental metals using the CTD (CTD 2011), **(2)** filter the metal-associated CTD genes/proteins for biological function related to birth defects, and **(3)** predict biological pathways influencing metal-induced developmental disorders.

Supplemental Material, Figure S2

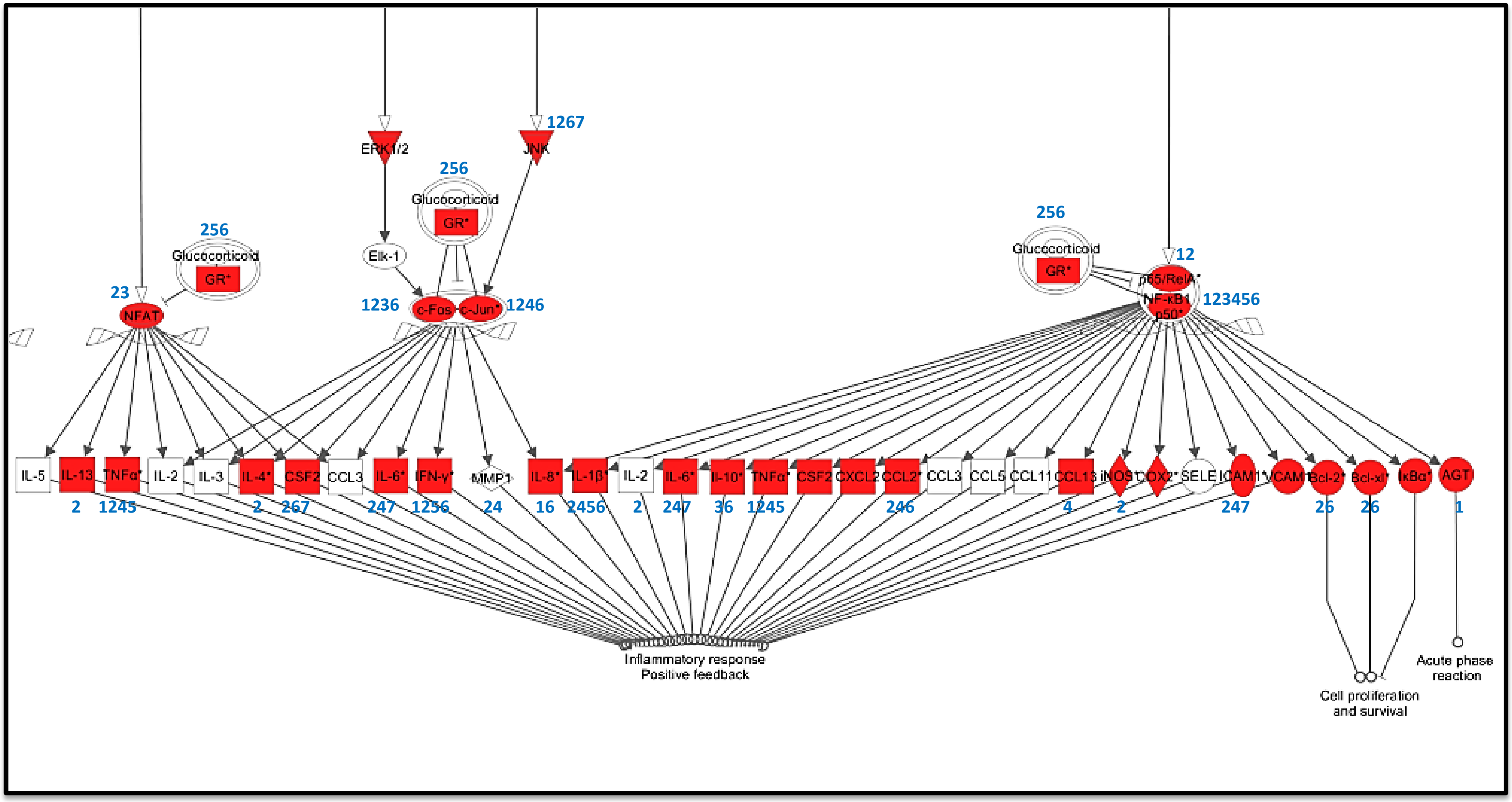

Supplemental Material, Figure S2: The glucocorticoid receptor (GR) signaling pathway is the most significant canonical pathway that associates metal exposure with birth defects. Network analysis highlights that most of the metals studied are associated with genes that encode proteins directly involved in the canonical GR signaling pathway. Red symbols represent proteins encoded by metal-associated genes involved in development. Numbers **1** through **7** represent each metal of study enriched for their involvement in the GR pathway: **(1)**: Cadmium ($CdCl_2$) (*p*-value 1.58×10^{-21}); **(2)**: Inorganic arsenic (iAs^{3+}) (*p*-value 1.91×10^{-50}); **(3)**: Mercury (Hg) (*p*-value 4.61×10^{-12}); **(4)**: Lead (Pb) (*p*-value 6.65×10^{-05}); **(5)**: Nickel (Ni) (*p*-value 1.86×10^{-15}); **(6)**: Selenium (Se) (*p*-value 2.7×10^{-23}); and **(7)**: Chromium (Cr) (*p*-value 8.8×10^{-11}). *p*-values represent the statistical significance of the enrichment for both metal and development associated genes in the GR pathway.

Supplemental Material, Figure S3

(A) **(B)**

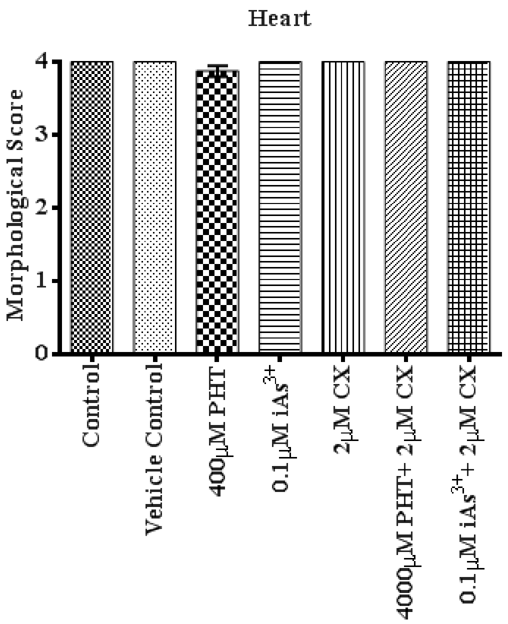

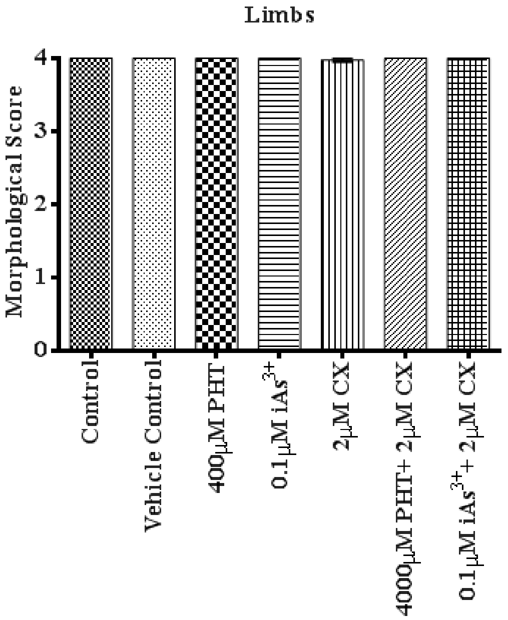

Supplemental Material, Figure S3: Morphological scores and embryonic growth. Chemical compound effects on embryonic development in whole chick embryo cultured *in ovo* (incubation day 3-6) on the **(A)** heart or **(B)** limbs. Data represent the averages from four independent experiments (mean ± S.E., n=4). **p*<0.05 compared with control and vehicle control groups. ***p*<0.01 compared with control and vehicle control groups. Abbreviations: Phenytoin (PHT); Inorganic arsenic (iAs^{3+}); Cortexolone (CX).

Supplemental Material, Figure S4

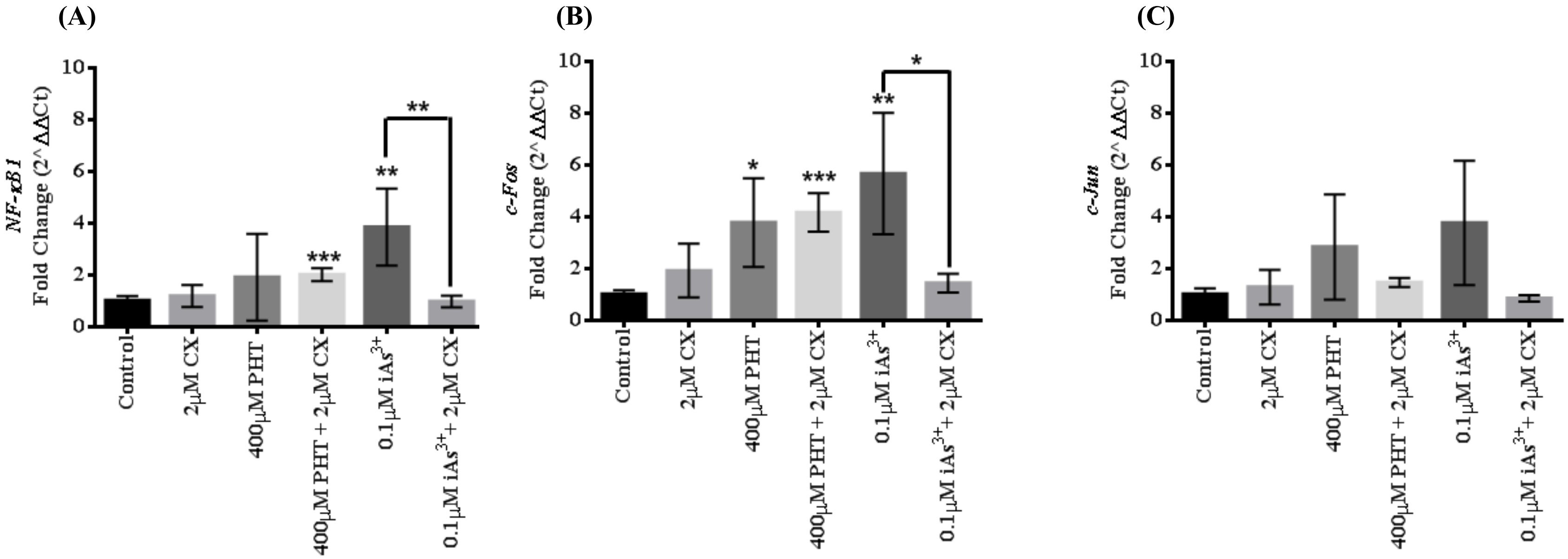

Supplemental Material, Figure S4: Quantitative real time PCR (q-RT-PCR) gene expression analysis of *NF-κB1* and *AP1* (comprised of *c-FOS* and *c-JUN*). Q-RT-PCR analysis for genes expression changes in chick embryo cultured *in ovo* exposed to inorganic arsenic (sodium arsenite) (incubation day 3-6) for **(A)** *NF-κB1*, **(B)** *AP1 (c-FOS)*, and **(C)** *AP1 (c-JUN)*. q-RT-PCR results displaying fold change gene expression (mean±S.E., n=3 embryos/group). *p*-values were calculated between exposure groups and control, or between exposure groups with or without cortexolone and are : *$p<0.05$, **$p<0.01$, or ***$p<0.001$ compared with control. Abbreviations: Phenytoin (PHT); inorganic arsenic (iAs^{3+}); Cortexolone (CX).

Supplemental Material, Table S1: Morphological scoring system based on Hamburger & Hamilton staging system (Hamburger and Hamilton 1951) (adapted and modified from Memon and Pratten 2009).

Embryonic feature	0	1	2	3	4
**Vitelline circulation**	No visible vessels	2–3 vessels	5–6 large vitelline vessels	Extensive network of vessels	Entire vitelline membrane well supplied
**Flexion**	None	Cranial flexure visible	Trunk rotation in addition to cranial flexion visible	Trunk rotation and tail bud rotation visible	Cranial flexure, trunk rotation and tail bud bending visible
**Heart**	No beating	Paired heart primordia	Beating heart bent to right	Atrioventricular canal and ventricular loop	Four-chambered appearance
**Brain**	Primitive streak	Neural folds visible	Brain vesicle, Anterior neuropore closed	Telencephalon enlarged, Forebrain forms right angle with hindbrain	Enlarged and transparent, Forebrain parallel to hindbrain
**Gross Facial Deformities**	None	Primary optic vesicles	Optic vesicles constricted at base	Optic cup completely formed on one side	Optic cup completely formed on both sides + beak is fully formed
**Limbs**	None	None	None	Digits and toes visible	Digits and toes visible on both sides

Supplemental Material, Table S2: List of primers for quantitative real-time RT-PCR analysis.

mRNA Target	Primer sequence
GAPDH	F 5'-AGTCCAAGTGGTGGCCATCAATG-3'<br>R 5'-TTAGCACCACCCTTCAGATGAGC-3'
c-FOS	F 5'-CATCTGTGAGAGCTGGTAGTCT-3'<br>R 5'- CCAGCACCAGGTTAATTCCAATCA-3'
c-JUN	F 5'- TGACTGGAAAGATGGAAACG-3'<br>R 5'- CCGTTGCTGGACTGGATTAT-3'
NF-κB1	F 5'-GAAGGAATCGTACCGGGAACA-3'<br>R 5'-CTCAGAGGGCCTTGTGACAGTAA-3'

Supplemental Material, Table S3: Selected metals of study.

Selected Metal Exposure	CAS Registry Number	CTD Genes (#)	Development-associated Genes (#)
**(a) Metal**			
**Arsenic (iAs^{3+})**	13768-07-5	1880	604
**Cadmium ($CdCl_2$)**	10108-64-2	518	167
**Chromium (Cr)**	7440-47-3	175	76
**Lead (Pb)**	7439-92-1	774	114
**Mercury (Hg)**	7439-97-6	334	104
**Nickel (Ni)**	7440-02-0	637	228
**Selenium (Se)**	7782-49-2	1616	370
**(b) Positive Control**			
**Phenytoin (PHT)**	57-41-0	138	79

http://dx.doi.org/10.1289/ehp.1205659

Supplemental Material, Table S4: The CTD curated metal-interacting genes/proteins.
Please see separate Excel file "12-05659-ART_Supplemental_Material_Table_S4"

Supplemental Material, Table S5: The genes that were identified as metal-associated and involved in development.

Please see separate Excel file "12-05659-ART_Supplemental_Material_Table_S5"

 http://dx.doi.org/10.1289/ehp.1205659

Supplemental Material, Table S6: p-values of the enrichment of the top five canonical pathways associated with each designated metal and phenytoin.
Please see separate Excel file "12-05659-ART_Supplemental_Material_Table_S6"

REFERENCES

CTD. 2011. CTD^{TM} Comparative Toxicogenomics Database. Available: http://ctdbase.org [accessed 1st September 2011].

Hamburger V, and Hamilton HL. 1951. A series of normal stages in the development of the chick embryo. J Morph 88:49-58.

Memon S, and Pratten MK. 2009. Developmental toxicity of ethanol in chick heart in ovo and in micromass culture can be prevented by addition of vitamin C and folic acid. Reprod Toxicol 28:262-269.
